# Patterns of Abundance, Host Use, and Everglades Virus Infection in *Culex* (*Melanoconion*) *cedecei* Mosquitoes, Florida, USA

**DOI:** 10.3201/eid2506.180338

**Published:** 2019-06

**Authors:** Isaiah J. Hoyer, Carolina Acevedo, Keenan Wiggins, Barry W. Alto, Nathan D. Burkett-Cadena

**Affiliations:** University of Florida, Vero Beach, Florida, USA

**Keywords:** Everglades virus, *Culex cedecei*, blood meal, vector-borne infections, zoonoses, viruses, Florida, United States, mosquitoes, hosts

## Abstract

Everglades virus (EVEV), subtype II within the Venezuelan equine encephalitis (VEE) virus complex, is a mosquitoborne zoonotic pathogen endemic to south Florida, USA. EVEV infection in humans is considered rare, probably because of the sylvatic nature of the vector, the *Culex* (*Melanoconion*) *cedecei* mosquito. The introduction of *Cx. panocossa*, a tropical vector mosquito of VEE virus subtypes that inhabits urban areas, may increase human EVEV exposure. Field studies investigating spatial and temporal patterns of abundance, host use, and EVEV infection of *Cx. cedecei* mosquitoes in Everglades National Park found that vector abundance was dynamic across season and region. Rodents, particularly *Sigmodon hispidus* rats*,* were primary vertebrate hosts, constituting 77%–100% of *Cx. cedecei* blood meals. Humans were fed upon at several locations. We detected EVEV infection in *Cx. cedecei* mosquitoes in lower and upper regions of Everglades National Park only during the wet season, despite an abundance of *Cx. cedecei* mosquitoes at other sampling times.

Everglades virus (EVEV) is a mosquitoborne alphavirus endemic to the state of Florida, USA, and is subtype II within the Venezuelan equine encephalitis (VEE) complex (*1*). The mosquito *Culex* (*Melanoconion*) *cedecei* is the sole enzootic vector of EVEV (*2**–**4*); rodents, particularly *Peromyscus gossypinus* (cotton mouse) and *Sigmodon hispidus* (hispid cotton rat), are the primary reservoir hosts (*5**–**7*). Clinical cases of EVEV infection in humans are considered rare; symptoms consist of nonspecific influenza-like febrile illness that can culminate in serious neurologic damage (*1*). The recent introduction and establishment of *Cx. panocossa* mosquitoes into Florida (*8*) could increase human exposure to EVEV because this species is a vector of endemic VEEV strains in Central America (*9**,**10*) and is abundant in manmade waterways supporting water lettuce (*8**,**11*). Studies in Panama concluded that *Cx. panocossa* (as *Cx. aikenii*) mosquitoes were the most important VEE vector on the basis of high VEE experimental transmission rates (*9*), high experimental infection rates (*9*), high population density (*9*), and feeding upon VEE reservoir hosts (*10**,**11*). The establishment of *Cx. panocossa* mosquitoes in urban areas could link sylvatic transmission foci of EVEV with densely populated areas such as the greater Miami metropolitan area through vegetated canals (*8*).

Evidence of sporadic human infections with EVEV in south Florida in the 1960s (*12**,**13*) spurred numerous field and laboratory studies to investigate the natural transmission cycle of the virus, focusing on determining the natural vectors and reservoirs of EVEV. These studies concluded that *Cx. cedecei* mosquitoes transmit EVEV between the amplifying rodent hosts (cotton mouse and hispid cotton rat) (*2**–**6*) in hammocks of the Greater Everglades ecosystem. Although EVEV vector and reservoirs were firmly incriminated, researchers repeatedly encountered unexplained large heterogeneity in EVEV transmission, even at very small scales, in Florida. For example, Chamberlain et al. (*2*) found that EVEV infection rates in vectors ranged from 0.18% (n = 533) to 1.7% (n = 58) in *Culex* (*Melanoconion*) spp. mosquitoes at 3 of 4 Everglades research areas in 1963 and 1964, but the virus was not recovered from Pa-hay-okee Overlook, despite relatively high exposure rates (20%) in rodents at that site (*2*). At Mahogany Hammock, 12 EVEV isolations were made from *Culex* (*Melanoconion*) females (average infection rate 0.53%), even though exposure rates in rodents at Mahogany Hammock were lower than at Pa-hay-okee (*2*). In subsequent field studies north of Everglades National Park (ENP), Bigler et al. (*14*) demonstrated that EVEV appeared and circulated at different time periods during the year at 2 hammocks, despite their similarity and proximity (<3 km). Although the density of cotton mice varied between hammocks, the populations and seasonal fluctuations in age ratios and breeding activity were comparable (*14*). The moderate abundance and low levels of EVEV activity in vectors led the researchers to hypothesize that mechanisms other than host densities, populations, age structure, and mosquito infection all contributed to fluctuations of EVEV transmission between the 2 hammocks (*14*).

We conducted our study to quantify the spatial and seasonal patterns of abundance, host use, and EVEV infection of *Cx. cedecei* mosquitoes in ENP to explore potential explanations for heterogeneity in EVEV prevalence observed in prior studies. We aspirated resting adult mosquitoes from 15 locations along ≈50 km of Main Park Road of ENP, where endemic EVEV transmission has been demonstrated in past studies. We used PCR assays to quantify host associations and EVEV infection in the mosquitoes and used logistic regression to investigate associations between EVEV infection, vector abundance, and host use.

## Methods

**Figure 1:**
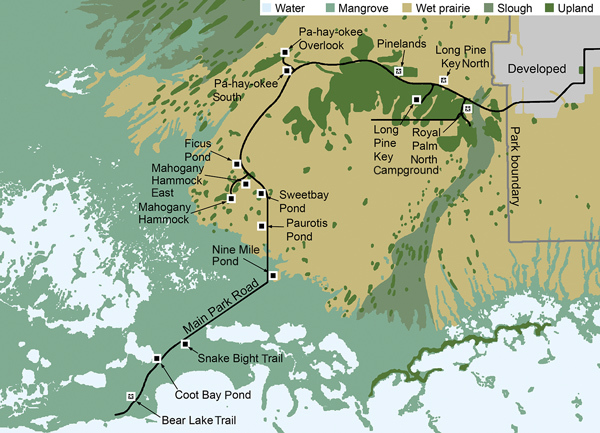
Everglades National Park, Florida, USA, showing dominant habitat types and sampling sites along Main Park Road. Black lines indicate paved roadways. Black squares indicate sampling sites; asterisks (*) within black squares denote detections of Everglades virus RNA in pooled *Culex cedecei* females by quantitative reverse transcription PCR.

We sampled mosquitoes in a variety of habitats along Main Park Road from the Everglades Visitor Center to Flamingo (permit EVER-2015-SCI-0054). The sampling locations were divided into 3 regions, upper, middle, and lower, representing natural physiographic regions of south Florida (Table 1; Figure 1). The upper region, from Royal Palm North (25°24′08.3′′N, 80°36′56.7′′W) to Pa-hay-okee South (25°25′56.0′′N, 80°46′38.9′′W), was dominated by large expanses of upland pine or hardwood forest. The middle region, from just south of Pa-hay-okee South extending to Nine Mile Pond (25°15′14.1′′N, 80°47′53.5′′W), was dominated by wet sawgrass prairie (Everglades marsh) with smaller hardwood hammocks (tree islands). The lower region, from Snake Bight Trail to Bear Lake Trail (25°8′55.82′′N, 80°55′23.69′′W), was dominated by extensive mangrove swamp. 

**Table 1:** Collection of female *Culex cedecei* mosquitoes by sampling site, Everglades National Park, Florida, USA, 2016

Region	Site name and coordinates	Habitat	Mos. sampled	No. resting shelter days*	Total no. females	No. females/shelter-day
Upper	Royal Palm North, 25°24′7.43′′N, 80°36′56.40′′W	Extensive hardwood hammock	Feb, May, Jun, Aug	60	810	13.50
	Long Pine Key North, 25°25′3.00′′N, 80°38′20.00′′W	Small island hammock	Jun, Aug	8	147	18.38
	Long Pine Key Campground, 25°24′0.10′′N, 80°39′35.40′′W	Extensive pine rockland	Feb	4	5	1.25
	Pinelands, 25°25′24.80′′N, 80°40′47.00′′W	Extensive pine rockland	May, Jun, Aug	44	107	2.43
	Pa-hay-okee South, 25°25′56.00′′N, 80°46′38.90′′W	Small island hammock	Feb, May	18	1	0.06
	Pa-hay-okee Overlook, 25°26′27.20′′N, 80°47′1.60′′W	Large island hammock	Jun, Aug	24	24	1.00
Middle	Ficus Pond, 25°21′24.00′′N, 80°49′20.00′′W	Small island hammock	Jun, Aug	8	47	5.88
	Mahogany Hammock East, 25°20′20.00′′N, 80°49′4.80′′W	Small island hammock	Feb, May, Jun, Aug	50	287	5.74
	Mahogany Hammock,† 25°19′22.50′′N, 80°49′59.40′′W	Large island hammock	May, Jun	4	2	0.50
	Sweet Bay Pond, 25°19′55.00′′N, 80°48′10.00′′W	Small island hammock	Jun, Aug	8	16	2.00
	Paurotis Pond, 25°18′7.00′′N, 80°47′56.00′′W	Small island hammock	Jun, Aug	8	42	5.25
	Nine Mile Pond, 25°15′13.93′′N, 80°47′53.64′′W	Ecotone of prairie and mangrove	Feb, May, Jun, Aug	53	942	17.77
Lower	Snake Bight Trail, 25°11′59.87′′N, 80°52′27.08′′W	Mangrove swamp	Feb, May, Jun, Aug	53	702	13.25
	Coot Bay Pond, 25°10′56.90′′N, 80°53′51.80′′W	Mangrove swamp	Feb	6	28	4.67
	Bear Lake Trail, 25° 8′55.82′′N, 80°5523.69′′W	Extensive cottonwood hammock	Feb, May, Jun, Aug	58	253	4.36
Totals				406	3,413	6.40

*Resting shelter days is no. of resting shelters deployed × total no. days sampled. †Natural aspirations were performed to abide by permit restrictions.

We aspirated all mosquitoes from resting shelters (*15*), except at Mahogany Hammock, where we targeted natural resting sites, such as fallen logs and deep recesses, to abide by permit restrictions. We performed aspirations using a modified handheld battery-powered vacuum (DustBuster BDH1800S; Black and Decker Corporation, https://www.blackanddecker.com) fitted with a funnel and stainless-steel mesh-bottom collection cup (BioQuip model 2846D; BioQuip Products, Inc., https://www.bioquip.com), as described previously (*16**,**17*). Resting shelters were equivalent in size and shape to previous models (*15*) but were constructed of PVC pipe and fittings so that they could be easily disassembled after each collecting trip, in accordance with permit requirements. We sampled a total of 406 resting shelter days in ENP, at a rate of 14–27 resting shelters per sampling period; we placed 1–5 shelters at each site and sampled them for 3–5 consecutive days between 7:00 am and 1:00 pm. We placed resting shelters in areas with maximum shade within 90 m of Main Park Road or 45 m of trails. Sampling occurred within the Everglades dry season (in February and May) and wet season (in June and August) of 2016.

We identified mosquitoes to species using morphological features of the adult (*16*). Because of well-known difficulties of identifying *Culex* (*Melanoconion*) females (*17**,**18*), we initially confirmed identifications by morphology of the cibarial armature (*17*) and molecular assays targeting the 18s mitochondrial gene (*18**)*. Narrow decumbent scales of the vertex (*19*) served as a helpful diagnostic feature to separate *Cx. cedecei* from other *Culex* (*Melanoconion*) spp. in Florida. 

We performed blood meal analysis on individual blood-engorged *Cx. cedecei* females using published PCR-based techniques (*20*). We amplified extracted DNA using PCR assays targeting cytochrome B and 16s rRNA genes of vertebrate hosts. We initially screened samples to identify blood meals from mammalian and amphibian hosts, using primers L2513/H2714 (*21*) that target 16S rRNA of the host animal (*20*). We then screened samples that produced no amplicon using primer pairs L0/H1 and/or L0/H0, both targeting the cytochrome b gene of birds (*22*), and primer pair 16L1/H3056 used in phylogenetic studies of reptiles (*23**,**24*). Primer sequences and cycling conditions are described in Blosser et al. (*20*). We sent PCR products to Eurofins Scientific (https://www.eurofins.com) for Sanger sequencing (forward direction only). We aligned sequences with published sequences in the National Center of Biotechnology Information sequence database using BLAST (https://blast.ncbi.nlm.nih.gov/Blast.cgi). We considered nucleotide similarity >95% a positive match. We maintained cold chain for all samples from the time of capture until DNA/RNA extraction. To minimize host DNA cross contamination during sorting and processing, we handled specimens by their legs (to avoid puncturing the abdomen) using clean forceps and gloved hands. In general, blood-engorged females from resting shelters were fully intact, with no evidence of ruptured abdomens. We discarded any specimen with ruptured abdomen.

We screened RNA extracts (QIAamp viral RNA mini kit; QIAGEN, https://www.qiagen.com) from pooled *Cx. cedecei* females for presence of EVEV RNA using quantitative reverse transcription PCR (qRT-PCR) assay with (5′→3′) primers (forward: CGAGGAGCTGTTTAAGGAGTATAA; reverse: CCTCTATGGCTATTGGGCTATG) and probe (6-FAM/CGTTAGGTGTGCCGTTGGGAGTT/3BHQ1/) targeting EVEV nucleocapsid structural protein (primers/probes design by Integrated DNA Technologies, https://www.idtdna.com). Cycling conditions were 50°C (30 min), 95°C (2 min), then 40 cycles of 95°C (15 s) and 61°C (1 min). For assay validation we used EVEV strain FE3-7C, obtained from the Centers for Disease Control and Prevention, propagated on Vero cells using standard techniques (*25**,**26*). Mosquito pools consisted of <32 unfed and gravid *Cx. cedecei* females aggregated by month and site. We determined samples with C_t_ values <33 to be positive for EVEV RNA.

### Statistical Analysis

We used Poisson regression modeling (*27**,**28*) to test for statistically significant differences in mosquito abundance between wet and dry seasons for each of the most commonly collected mosquito species in each region, using Akaike information criterion (AIC) and the ratio of the Poisson regression coefficients to the SE (z) as test statistics. We calculated mosquito abundance as the number of females per shelter per sampling day. Only sites that were sampled during all sampling months were included in the analysis: Royal Palm North and Pinelands (upper), Mahogany Hammock East and Nine Mile Pond (middle), and Snake Bight Trail and Bear Lake Trail (lower). We performed χ^2^ test of independence to determine whether *Cx. cedecei* blood meals were distributed differently among host species between season and region. Using multiple logistic regression, we determined whether vector abundance or reservoir host use better predicted detection of EVEV RNA from pooled mosquitoes. We quantified reservoir host use as the number of hispid cotton rat or cotton mouse blood meals per resting shelter per day. We performed statistical tests using R Studio version 3.4.3 (https://www.rstudio.com) and SAS version 9.4 (https://www.sas.com). For all statistical tests, α = 0.05.

## Results

**Figure 2:**
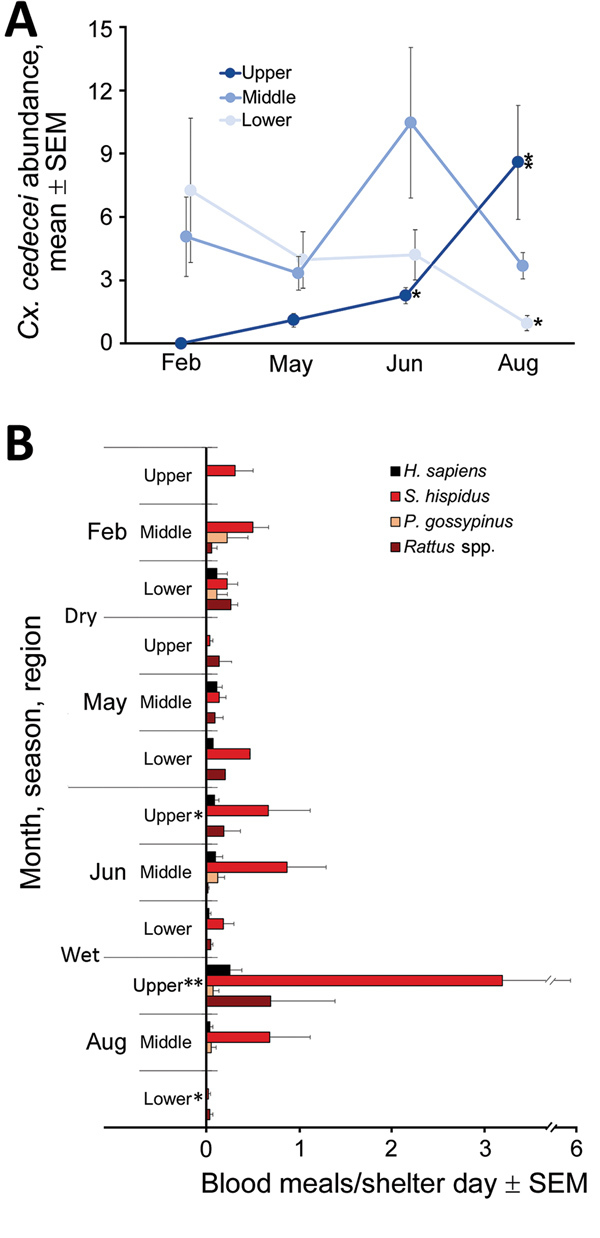
Regional and seasonal abundance and host use of *Culex cedecei* mosquitoes in Everglades National Park, Florida, USA. Wet season is April–October, and dry season is November–March. Each asterisk (*) denotes a pool of *Cx. cedecei* females that tested positive for Everglades virus RNA by quantitative reverse transcription PCR. A) Average number of females aspirated from resting shelters by month. B) Host use by *Cx. cedecei* mosquitoes, represented as blood meals per resting shelter day by region and season.

*Cx. cedecei* mosquito abundance varied across season and region (Figure 2, panel A). Females were significantly more abundant in the wet season in upper (AIC = 228.22, z = 6.488; p<0.001) and middle (AIC = 295.24, z = 3.811; p<0.001) regions of the Everglades but significantly more abundant during the dry season in the lower region (AIC = 251.2, z = −3.897; p<0.001). No other mosquito species exhibited a pattern of greater abundance during the dry season in any region (Appendix Table 1). For all other commonly collected mosquito species, abundance was greater during the wet season than dry season in all areas, although differences were not always significant (Appendix Table 1). Even within a single region and sampling period, *Cx. cedecei* mosquito abundance varied considerably. For example, we collected 13.50 females per resting shelter at Royal Palm North (60 shelter days), whereas we collected 2.43 females per resting shelter at Pinelands (44 shelter days); the distance between the sites was only ≈6.7 km.

The distribution of host species blood meals was significantly different spatially (χ^2^ = 90.90, df = 20; p<0.001) between the lower, middle, and upper regions of ENP (Figure 2, panel B) but not seasonally (χ^2^ = 14.49, df = 10; p = 0.152) (Figure 2, panel B). Rodents accounted for 77%–100% of the 347 total identifiable blood meals (77.0% of 451 samples returning >95% match), depending on season and region (Table 2). Blood meals from EVEV reservoirs (hispid cotton rat and cotton mouse combined) were a large percentage of total blood meals from all regions, constituting 48% of total blood meals from the lower, 84% from the middle, and 50% from the upper ENP region (Table 2). Reservoir host blood meals originated overwhelmingly from hispid cotton rat (Table 2; Figure 2, panel B), regardless of region or season. However, cotton mouse blood meals were relatively more common in the dry season in the lower and middle ENP (Figure 2, panel B). Blood meals from invasive *Rattus* spp. rodents contributed to the significant difference in distribution of host blood meals by region, such that much higher numbers of *Rattus* spp. blood meals were encountered in upper (40%) and lower (30%) than middle (2%) regions of ENP (Figure 2, panel B). Blood meals obtained from humans constituted 7.53% of total blood meals and were detected in all 3 regions and seasons. All other hosts constituted <1% of *Cx. cedecei* host blood meals (Table 2; Appendix Table 2).

**Table 2:** *Culex cedecei* mosquito blood meal host species, by park region and season, Everglades National Park, Florida, USA, 2016

Vertebrate host species	Lower			Middle			Upper			Total
	Dry	Wet		Dry	Wet		Dry	Wet		
*Anolis sagrei*	0	0		0	3		0	0		3
*Homo sapiens sapiens*	6	1		2	9		0	16		34
*Lontra canadensis*	0	0		0	0		0	1		1
*Neotoma floridana*	0	0		0	0		0	1		1
*Odocoileus virginianus*	1	0		0	1		0	1		3
*Oryzomys palustris*	0	1		0	1		0	0		2
*Peromyscus gossypinus*	3	0		4	6		0	1		14
*Procyon lotor*	1	1		0	0		0	0		2
*Rattus* spp.	12	3		1	1		5	52		85
*Sigmodon hispidus*	12	9		21	73		5	81		201
*Sylvilagus floridanus*	0	0		1	0		0	0		1
Undetermined	6	5		9	53		2	29		104
Total	41	20		38	147		12	193		451

Although the distribution of blood meals from different host species did not vary substantially between seasons, the number of blood meals from reservoir hosts by season and region did (Figure 2, panel B). The number of hispid cotton rat–fed females per resting shelter day was greatest in the wet season in the upper region, peaking at 3.20 hispid cotton rat blood meals per shelter-day in August in the upper region. By contrast, we encountered 0.02 hispid cotton rat blood meals per shelter-day in lower ENP that same month (Figure 2, panel B).

In total, we screened 3,673 females (1,326 in dry season, and 2,347 in wet season) for EVEV RNA by RT-PCR (average pool size + SD of 22 + 6 females) (Table 1), including 3,413 females from resting shelters and 260 females aspirated from natural resting sites. We found 4 pools of *Cx. cedecei* females, all of which were from the wet season (June or August), to be positive for EVEV by RT-PCR. These EVEV-positive pools included 1 pool of 25 females from Pinelands (upper region) in June (C_t_ = 21.3), 1 pool of 8 females from Bear Lake Trail (lower region) in August (C_t_ = 21.8), 1 pool of 25 females from Royal Palm North (upper region) in August (C_t_ = 24.8), and 1 pool of 25 females from Long Pine Key North (upper region) in August (C_t_ = 24.5). Multiple logistic regression showed no association between EVEV RNA detected in pooled *Cx. cedecei* and *Cx. cedecei* abundance, reservoir (hispid cotton rat plus cotton mouse) host use, or proportion of blood meals from reservoir hosts (χ^2^ = 4.08, df = 3; p = 0.252).

## Discussion

The dynamic nature of *Cx. cedecei* mosquito abundance is probably shaped by seasonal fluctuations in water levels through the seasonal filling and depletion of limestone solution holes (pits in karst that formed when sea level was lower than present levels), which constitute the primary habitat of *Cx. cedecei* larvae (*29*). The contrasting patterns of *Cx. cedecei* abundance in lower and upper regions of ENP suggest that Everglades hydrology has a strong influence on the seasonal reproductive biology of *Cx. cedecei* mosquitoes, which may have consequences for EVEV transmission. In addition, the finding that *Cx. cedecei* was the only mosquito species more abundant in dry season in the lower region of ENP suggests that the positive association between precipitation and abundance of other mosquito species may not apply consistently for this species. Our study did not quantify precipitation nor the availability of larval habitat, so explanations of the links between season, region, and *Cx. cedecei* abundance are speculative. One possible explanation for the higher abundance of *Cx. cedecei* mosquitoes during the dry season in the lower region is seasonal drying in the low-elevation lower Everglades: during times of higher water, aquatic predators, particularly fish, disperse rapidly throughout ENP (*30**,**31*), whereas isolated pockets of water without fish may be more abundant during the dry season. Detailed field and laboratory studies focusing on the ecology of immature stages are needed to understand the mechanisms driving these contrasting patterns of *Cx. cedecei* abundance throughout the Everglades.

Variation in *Cx. cedecei* host use between regions has important implications for understanding the transmission of EVEV in Florida. Our data demonstrate that *Cx. cedecei* mosquitoes feed heavily on mammals; rodents make up a substantial portion of hosts, regardless of season or region. The finding that hispid cotton rat and cotton mouse together constituted a large portion (43.0%–86.2%) of *Cx. cedecei* blood meals confirms a strong association between *Cx. cedecei* mosquitoes and these EVEV reservoir host species (*3**,**14*). Both hispid cotton rat and cotton mouse are common throughout Florida, so their role in EVEV transmission is probably limited by the distribution and abundance of *Cx. cedece* mosquitoes *i*. However, the introduction and establishment of *Cx. panocossa* mosquitoes in Florida may change this dynamic, resulting in more areas at risk for EVEV transmission (*8*). 

The importance of *Rattus* spp. rodents as hosts of EVEV in lower and upper regions of ENP bears additional investigation, because these rats were relatively common hosts of *Cx. cedecei* mosquitoes in these regions (20.0%–55.5% of total). We expect the lower and upper regions of the park, because of their proximity to park boundaries, campgrounds, parking lots, and human activity, to host larger populations of invasive rats than the middle region of ENP, which is comparatively undisturbed. Little information is available on the importance of *Rattus* spp. rodents as hosts of EVEV. Sanmartin et al. (*32*) made 4 isolations of VEEV from 41 *Rattus* spp. rodents sampled during an epizootic of VEEV in El Carmelo, Colombia, where subtypes IAB and IC circulate. In Florida, Bigler (*33*) detected EVEV antibodies in 12.5% (n = 40) *R. rattus* rats sampled east of ENP. These findings suggest that these widespread, invasive rats might also support the transmission of EVEV in Florida, although laboratory host competence studies are needed to address this hypothesis. 

The regions of the park in which human footprint is greatest also had the greatest relative numbers of human blood meals (11.48% lower; 7.80% upper), compared with findings from the middle region (5.95%). These relatively high levels of feeding suggest that *Cx. cedecei* mosquitoes could serve not only as an enzootic and epizootic vector but also as a potential epidemic vector of EVEV where it comes into contact with humans.

The lack of a clear association between EVEV in pooled *Cx. cedecei* females and vector abundance or metrics of host use is perplexing. Three of 4 EVEV-positive pools were from upper ENP during the wet season (1 in June, 2 in August), when *Cx. cedecei* mosquitoes were more abundant (Figure 2, panel A) and obtained a large number of blood meals from cotton rats (Figure 2, panel B). Conversely, the EVEV-positive pool from lower ENP was also from the wet season (August), but *Cx. cedecei* mosquito numbers were relatively low (Figure 2, panel A), and we observed very few blood meals from rodents (Figure 2, panel B). It is possible that wet-season transmission of EVEV is driven largely by the ecology and reproductive biology of cotton mouse and hispid cotton rat; however, a complex picture of rodent breeding and population dynamics emerges from past studies on this topic. Bigler et al. (*14*) concluded that the preponderance of EVEV amplification occurred between July and October, when dense populations of both cotton mice and cotton rats inhabited hammocks in the Pinecrest area on the ecotone between the Big Cypress Swamp and the Everglades ecosystem, ≈38 km northwest of the nearest sampling sites in our study. Lord et al. (*34*) screened mammals for EVEV antibodies in both Big Cypress Swamp and the Everglades, including the upper (Royal Palm Hammock) and middle (Mahogany Hammock) regions sampled in our study. Those results indicated that rodent breeding in these Everglades regions peaked in the dry season (January–February), a reversal of breeding patterns found farther north (*14*). Smith and Vrieze (*35*), working on hammocks of Taylor Slough (southeast Everglades), stated that all rodent reproduction occurred in the wet season. These contrasting results indicate that *S. hispidus* rats and *P. gossypinus* mice are likely to time their breeding to coincide with local conditions, which may be substantially different across locales. Our results are evidence of spatial variation in seasonality of EVEV transmission as observed in our work and that of others.

It was surprising that we did not detect EVEV in *Cx. cedecei* mosquito samples from the middle region of ENP, despite relatively high *Cx. cedecei* abundance (Figure 2, panel A), a relatively high biting rate on hispid cotton rats (Figure 2, panel B), and past evidence of EVEV circulation in mosquitoes in that region (*2*). Most *Cx. cedecei* females sampled from the middle region (70.5%; 942/1,336; Table 1) were captured at Nine Mile Pond on the ecotone between sawgrass marsh and mangrove swamps (Figure 1), a location for which historical data on EVEV prevalence is not available.

Although results of our work do not provide a complete understanding of EVEV transmission in ENP, they may help to clarify the perplexing heterogeneity observed in previous studies of EVEV in south Florida. The absence of EVEV in mosquitoes at Pa-hay-okee Overlook (*2*), for example, could be due to a near absence of *Cx. cedecei* mosquitoes at that site (Table 1). Differences in EVEV transmission at 2 adjacent hammocks observed by Bigler et al. (*14*) could be a result of differences in vector abundance, contact rates, or both between vectors and reservoir versus nonreservoir hosts, as observed in our study. We did not quantify the actual abundance of reservoir hosts in the field, so it is possible that a given set of mosquitoes all could have fed on a small number (in low population settings) or a large number (greater populations) of rodents. Our measure of host usage could not account for those differences, nor could it account for how many of those rodents were actually susceptible hosts. The number of available susceptible reservoir hosts is an important factor in maintaining enzootic cycling of viruses. Our data do support conclusions of past studies that EVEV transmission occurs seasonally and is heterogeneous across ENP. Relative number of feedings on reservoir hosts differs across regions of the park, indicating the vector infection ratio may be higher in specific habitats where *Cx. cedecei* mosquitoes have ample access to reservoir hosts, particularly young, virus-susceptible rodents.

Quantifying the spatial and temporal variation in abundance, host use, and virus infection of *Cx. cedecei* mosquitoes is a step toward understanding the ecology of EVEV transmission in the United States. Findings that *Cx. cedecei* mosquitoes feed on both reservoir hosts and humans in nature suggests that this insect could serve as both enzootic and epizootic vector. The establishment of the *Cx. panocossa* mosquito in Florida, and its putative spread, may change the spatial risk for EVEV transmission, if found to be a competent vector. Future work should evaluate the host competence of *Rattus* spp. rodents for EVEV and other VEEV subtypes, evaluate vector competence of *Cx. panocossa* mosquitoes for these viruses, and perform longitudinal studies of the focality of transmission in Florida.

Appendix Additional information about patterns of abundance, host use, and Everglades virus infection in *Culex* (*Melanoconion*) *cedecei* and other mosquitoes*,* Florida.
